# Preparation and Properties of UV-Curable Waterborne Polyurethane Acrylate/MXene Nanocomposite Films

**DOI:** 10.3390/nano13233022

**Published:** 2023-11-26

**Authors:** Ying Wang, Shuai Zhang, Yanli Lin, Qianyi Wang, Ying Zhang, Changmei Sun, Rongjun Qu

**Affiliations:** School of Chemistry and Materials Science, Ludong University, Yantai 264025, China; 23126248@bjtu.edu.cn (S.Z.); 20202403551@m.ldu.edu.cn (Y.L.); 2022110210@m.ldu.edu.cn (Q.W.); zhangying516@ldu.edu.cn (Y.Z.); sunchangmei0535@126.com (C.S.)

**Keywords:** waterborne polyurethane acrylate, MXene, UV-curable, nanocomposite films

## Abstract

In this study, waterborne polyurethane acrylate (WPUA)/MXene nanocomposite films with varying MXene loadings were fabricated using UV-curing technology, where MXene (Ti_3_C_2_T_x_) was employed as a nanofiller. The microstructure and chemical structure of the WPUA/MXene nanocomposite films were examined by XRD and FTIR, respectively. The water contact angle testing demonstrated that the incorporation of MXene into the nanocomposite films led to an increase in their hydrophilic properties. The tensile strength, the elongation at break, and Young’s modulus of the WPUA/MXene nanocomposite coatings exhibited an initial increase followed by a decrease with increasing MXene loadings. Compared to the pure WPUA film, the tensile strength and elongation at break of nanocomposites with 0.077 wt% MXene loading reached their maximum values, which increased by 39.9% and 38.5%, respectively. Furthermore, the glass transition temperature and the thermal stability were both enhanced by MXene to some extent. This study introduces a novel method for utilizing MXene in UV-curable waterborne coatings.

## 1. Introduction

UV-curing technology has the advantages of high efficiency, energy conservation, and eco-friendliness and has developed rapidly since it was commercialized. However, traditional UV-curable coatings often require the addition of active diluent monomers to adjust viscosity, and these active diluent monomers are mostly highly irritating and even harmful to the environment and human health. Waterborne UV-curable coatings combine the advantages of waterborne coatings and traditional UV-curable coatings. They consist of hydrophilic photosensitive resins as oligomers, waterborne photoinitiators, and various waterborne additives to form a coating formula. Due to the viscosity of the system being adjusted through water, there is no need to add active diluent monomers, making them more environmentally friendly and safer. In addition, waterborne UV-curable coatings have a low volume shrinkage after curing, with strong adhesion between the substrate and the film. Although they require the evaporation of water before UV-curing, increasing their energy consumption, their mechanical strength, chemical resistance, and corrosion resistance are better than those of traditional waterborne coatings. Therefore, waterborne UV-curable coatings have become an important development direction in the coatings industry.

Waterborne polyurethane acrylate (WPUA) is an important type of waterborne photosensitive resin due to its outstanding comprehensive properties, such as excellent chemical and solvent resistance, high impact resistance, and excellent flexibility. However, it also has some limitations in applications, such as poor weather resistance and generally lower mechanical performance [1,2]. The addition of nanomaterials is a viable approach to enhance the properties of UV-curable WPUA and even provide new functions for films. For example, UV-curable WPUA films with the addition of silica dioxide not only exhibit improved hardness, wear resistance, and weatherability but also demonstrate excellent temperature sensitivity and reversibility, making them suitable for thermochromic coatings [1]. Chen et al. [2] prepared WPUA/cationic TiO_2_/reduced graphene oxide nanocomposites with good mechanical properties and the ability for self-cleaning. Wu et al. [3] incorporated antimony-doped tin oxide into WPUA coatings, resulting in good thermal insulation performance. In addition, the addition of graphene oxide [4], ZnO [5], octavinyl polyhedral oligomeric silsesquioxane [6], nano calcium carbonate [7], surface-modified gamma-Al_2_O_3_ [8], etc. can also effectively improve the performance of WPUA films.

MXene, as a member of the two-dimensional transition metal carbide or carbonitride family, was first found by Yury Gogotsi’s team in 2011 [9]. Its chemical structure is represented by M_n+1_X_n_T_x_, where M represents a transition metal, X represents C or/and N, T represents surface termination functional groups (such as O^2−^, OH^−^, F^−^), and n = 1, 2, or 3. MXene exhibits good conductivity and mechanical properties, rich surface functional groups, and a high specific surface area, making it highly promising in the field of polymer nanocomposites. In recent years, MXene has been applied in various polymer matrices, such as epoxy resin [10,11,12], polyvinylidene fluoride [13,14], polyurethane [15,16], natural rubber [17], polyvinyl alcohol [18,19,20], polystyrene [21], etc. Researchers have reported that MXene can effectively enhance various properties of polymers, such as thermal stability and mechanical properties [10,16,20], conductivity and electromagnetic shielding performance [11,12,13,14,15,17,21], gas barrier properties [19], electro/photo-thermal conversion performance [13], heat dissipation performance [14], and flame retardancy [17]. To our knowledge, only Huang et al. [22] have incorporated MXene into a UV-curable coating system. They introduced surface-modified MXene into an intumescent flame-retardant (IFR) coating system, resulting in the fabrication of a UV-curable intumescent flame-retardant/MXene nanocomposite coating with excellent flame-retardant properties.

In this study, for the first time, MXene (Ti_3_C_2_T_x_) was introduced into a UV-curable WPUA coating system. On the one hand, the MXene solution can be directly added to the waterborne photosensitive resin to adjust the viscosity. On the other hand, compared with the solid form of MXene, MXene in solution form can be better mixed with the waterborne resin, which makes the two-dimensional MXene nanosheets have better dispersion in the polymer. The microstructure and chemical structure of the MXene as well as nanocomposite films were characterized. The influence of MXene on the hydrophilicity and mechanical properties of the coatings were individually studied through water contact angle measurements and tensile testing, respectively. The interface interaction of MXene and a polymer matrix was investigated by a cross-sectional scanning electron microscope (SEM). In addition, the influence of MXene on the glass transition temperature and thermal stability of the films was studied using differential scanning calorimetry (DSC) and thermogravimetric analysis (TGA), respectively. This study lays the foundation for the application of MXene in the field of UV-curable coatings.

## 2. Materials and Methods

### 2.1. Materials

HCl was supplied by Yantai Yuandong Fine Chemical Co., Ltd., Yantai, China. LiF was obtained from Sinopharm Chemical Reagent Co., Ltd., Shanghai, China. Ti_3_AlC_2_ powder was purchased from Ningbo Jinlei Nanomaterials Technology Co., Ltd., Ningbo, China. Waterborne polyurethane acrylate (JZ-4234) was obtained from Nanjing Jiazhong Chemical Technology Co., Ltd., Nanjing, China. Photoinitiators 2959 and TPO were both purchased from thunchem (Shanghai) Co., Ltd., Shanghai, China.

### 2.2. Synthesis of MXene

Add 3.2 g of LiF and 40 mL of 9 mol/L HCl solution to a polytetrafluoroethylene container. Stir the mixture using a magnetic stirrer for 20 min to dissolve LiF. Continue stirring and slowly add 2.0 g of Ti_3_AlC_2_. Ti_3_AlC_2_ was etched for 24 h under magnetic stirring at 45 °C. After the etching process is complete, centrifuge the solution at 4000 r/min until the supernatant reaches a neutral pH. Mix the collected precipitate with a certain amount of deionized water and transfer it into a flask. Inject nitrogen gas and ultrasonicate the mixture in an ice-water bath for 2 h at a frequency of 40 kHz. At last, after centrifugation at 4500 rpm for 40 min, the upper layer of the MXene solution was collected.

Take a clean beaker and weigh it. Using a pipette, transfer a certain amount of MXene solution into the beaker. After drying at 60 °C for 18 h under vacuum, weigh the beaker again. Calculate the concentration of the MXene solution to be 7.7 mg/mL.

### 2.3. Preparation of UV-Curable WPUA/MXene Nanocomposites

Take a certain amount of WPUA and add 3 wt% of photoinitiator 2959 and 1 wt% of TPO. Heat the mixture at 50 °C in a water bath while stirring until the photoinitiator is completely dissolved. Take a certain amount of the aforementioned UV-curable WPUA coating and add a specific quantity of the MXene solution and deionized water. Stir well to obtain UV-curable WPUA/MXene nanocomposite coatings with MXene content of 0, 0.077, 0.154, 0.213, and 0.308 wt%, respectively, denoted as WPUA/MXene-0, WPUA/MXene-1, WPUA/MXene-2, WPUA/MXene-3, and WPUA/MXene-4. The specific formulations are shown in Table 1.

**Figure 1:**
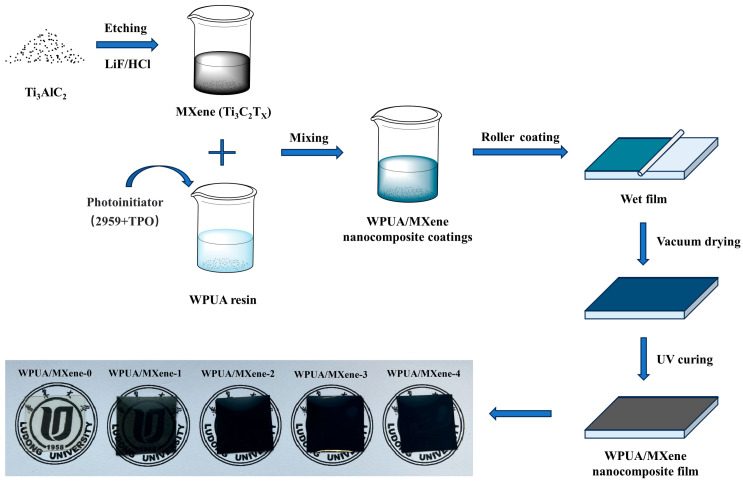
Schematic illustration of the fabrication process for the WPUA/MXene nanocomposite films.

The coating was coated on a glass plate using an RDS 225G# 3/8″ bar coater (R. D. SPECIALTIES Inc., Odessa, TX, USA. and an OCEAN SCIENCE 411 coating machine (OCEAN SCIENCE Co., Ltd., Seoul, Republic of Korea), and the thickness of the wet film was 514.4 μm. Then, the coated glass plate was dried at 60 °C for 6 h under vacuum (−0.08 MPa). After that, UV curing was performed using a ZB300 UV curing equipment (Tai’an Zibor Photoelectric Technology Co., Ltd., Tai’an, China) with a Fusion 300 s UV light source (light intensity of 2.5 W/cm^2^) to obtain the WPUA/MXene nanocomposite film. The cured film had a thickness of approximately 250 μm. The schematic representation of the fabrication process can be seen in Figure 1.

### 2.4. Characterization

Transmission electron microscopy (TEM) analysis was conducted employing a Talos F200X G2 high-resolution field emission transmission electron microscope (Thermo Fisher Scientific Inc., Wilmington, MA, USA). XRD analysis was performed using a SmartLab SE X-ray diffractometer (Rigaku Corporation, Tokyo, Japan). The scanning range was set from 5° to 80° with a scanning speed of 5°/min. A copper target was used with a tube voltage of 40 kV and a current of 40 mA. FTIR spectroscopy was conducted using a Nicolet iS50 spectrometer (Thermo Fisher Scientific Inc., Wilmington, MA, USA). Water contact angle measurements were carried out using a POWEREACH JC2000D3M contact angle measurement instrument (Shanghai Zhongchen Digital Technology Equipment Co., Ltd., Shanghai, China). The tensile properties of the nanocomposites were evaluated using an Instron 5967 universal testing machine. The testing was conducted in accordance with the ASTM D882 standard [23]. The sample with an average thickness of around 250 μm was cut into a strip with a width of 10 mm and a length of 250 mm using a blade, and care was taken to avoid notches and tears on the specimen during cutting. The loading rate during testing was set at 50.0 mm/min with a gauge length of 100 mm. Five parallel tests were conducted for each component of the sample. The fracture surface morphology of the film was characterized using a TESCAN MIRA LMS scanning electron microscope (TESCAN, Brno, Czech). The film was fractured under liquid nitrogen and coated with a layer of gold using a sputtering technique. DSC testing was performed using a TA Q2000 differential scanning calorimeter (TA Instruments, New Castle, Pennsylvania, USA). The temperature range of the analysis was set from −80 to 200 °C, and the heating rate was maintained at 10 °C/min. The glass transition temperature (T_g_) is determined by the appearance of a step in the first heating curve that changes direction toward an endothermic direction. The temperatures corresponding to the intersection points of the baseline extension lines and the tangent line to the curve at the step are considered as the starting and ending points of the glass transition. The average of these two temperatures is defined as the T_g_. TGA was performed using a TA TGA55 analyzer (TA Instruments, New Castle, PA, USA). The analysis was conducted in a nitrogen atmosphere, with a heating rate of 10 °C/min from room temperature to 700 °C.

## 3. Results and Discussion

### 3.1. Characterization of MXene

**Figure 2:**
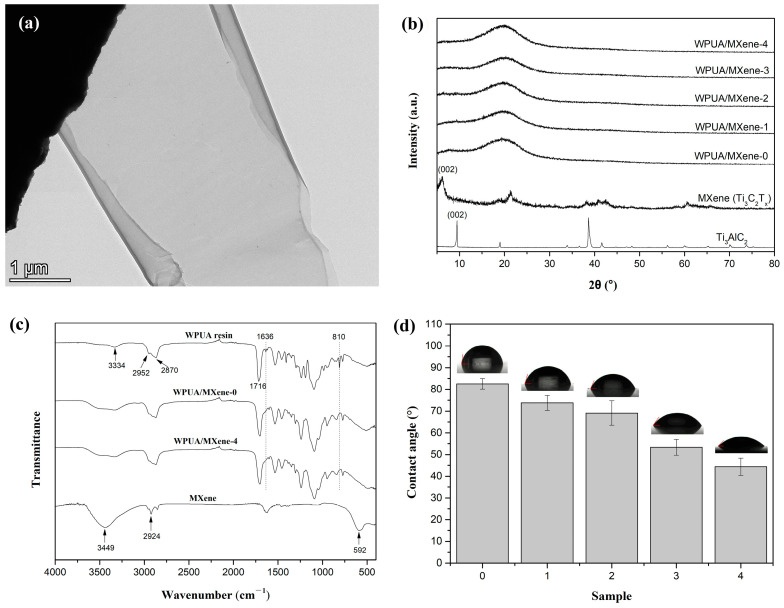
(**a**) TEM micrograph of MXene, (**b**) XRD patterns of MXene and WPUA/MXene nanocomposite films, (**c**) FTIR spectra of the MXene, uncured WPUA resin, WPUA/MXene-0 and WPUA/MXene-4 films, and (**d**) water contact angle of WPUA/MXene nanocomposite films.

Figure 2a demonstrates a representative TEM image of MXene, revealing its ultra-thin nanosheet morphology characterized by a transparent and distinctive two-dimensional layered structure. Figure 2b displays the XRD spectra of the raw material Ti_3_AlC_2_ and MXene (Ti_3_C_2_T_x_). The observed shift in the diffraction peak corresponding to the (002) crystal plane can be noticed, with Ti_3_AlC_2_ exhibiting a peak at 9.5° while MXene shows a peak at 6.2°, indicating the successful delamination of Ti_3_AlC_2_ to form MXene with larger interlayer spacing through etching [22,24,25].

### 3.2. Characterization of WPUA/MXene Nanocomposite Films

XRD analysis was conducted to investigate the microstructure of the WPUA/MXene nanocomposite films. In the XRD pattern of WPUA/MXene-0 film, only a broad peak appears around 2θ = 20°, indicating the amorphous structure of the polymer. The XRD peaks of the WPUA/MXene nanocomposite films are similar to those of the WPUA/MXene-0 film, without the characteristic peaks of MXene. This may be due to the small amount of MXene added and its uniform dispersion in the matrix, which is not sufficient to form a crystalline phase.

Furthermore, the chemical structures of MXene, the uncured WPUA resin, and the WPUA/MXene-0 and WPUA/MXene-4 films were characterized by FTIR (Figure 2c). The infrared spectrum of MXene shows a characteristic peak at 3449 cm^−1^, corresponding to the stretching vibrations of the –OH groups on the surface of the MXene nanosheets [22], as well as peaks at 2924 and 592 cm^−1^ that can be attributed to the stretching vibrations of C–H and Ti–O bonds, respectively [26,27]. In the infrared spectrum of the WPUA resin, peaks at 1716 and 3334 cm^−1^ are ascribed to the stretching vibrations of C=O and N–H bonds, respectively, while peaks at 2870 and 2952 cm^−1^ represent the characteristic vibrations of –CH_2_ and –CH_3_ groups, respectively, and the peak of the C=C bond can be found at 1636 and 810 cm^−1^ [3]. It can be observed from the infrared spectra of WPUA/MXene-0 and WPUA/MXene-4 films that the characteristic peaks of the C=C bond at 1636 and 810 cm^−1^ disappear completely, indicating that all the C=C bonds participate in the free radical photo-initiated polymerization reaction. It also confirms that the addition of MXene at a weight percentage of 0.308 wt% does not adversely affect the conversion rate of C=C bonds. In addition, the characteristic peaks of WPUA/MXene-4 are similar to those of WPUA/MXene-0, and no characteristic peaks of MXene are detected. This may be due to the overlap between the characteristic peaks of WPUA and MXene, as well as the low amount of MXene added, which is below the detection limit.

### 3.3. Hydrophilicity of WPUA/MXene Nanocomposite Films

The hydrophilicity of the WPUA/MXene nanocomposite films was investigated by measuring the contact angle between the water and the film (Figure 2d). The water contact angle of WPUA/MXene-0 was 82.5°. As the MXene loadings increased, a gradual decrease in the water contact angle of the nanocomposite films was observed. When the MXene content reached 0.308 wt% (WPUA/MXene-4), the water contact angle decreased to 44.4°. This demonstrates that the addition of MXene enhances the hydrophilicity of the nanocomposite films, primarily due to the inherently excellent hydrophilic properties of MXene nanosheets [17,28].

### 3.4. Mechanical Properties of WPUA/MXene Nanocomposite Films

**Figure 3:**
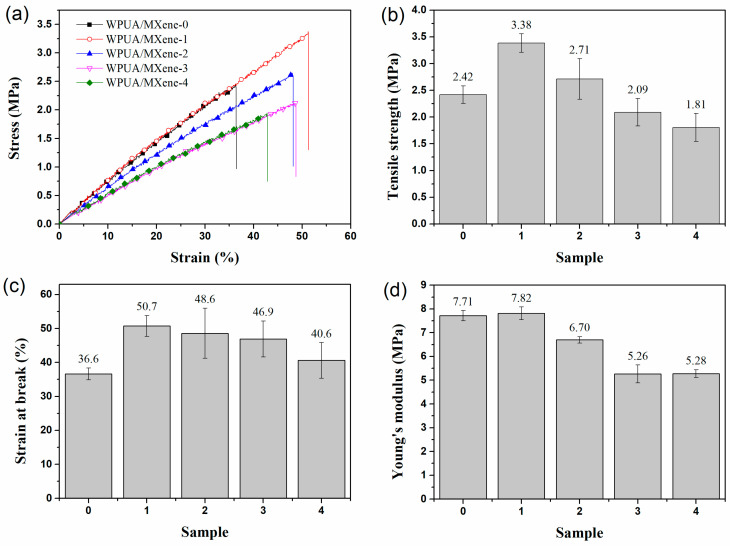
(**a**) Typical stress–strain curves, (**b**) tensile strength, (**c**) elongation at break, and (**d**) Young’s modulus of the WPUA/MXene nanocomposite films.

**Figure 4:**
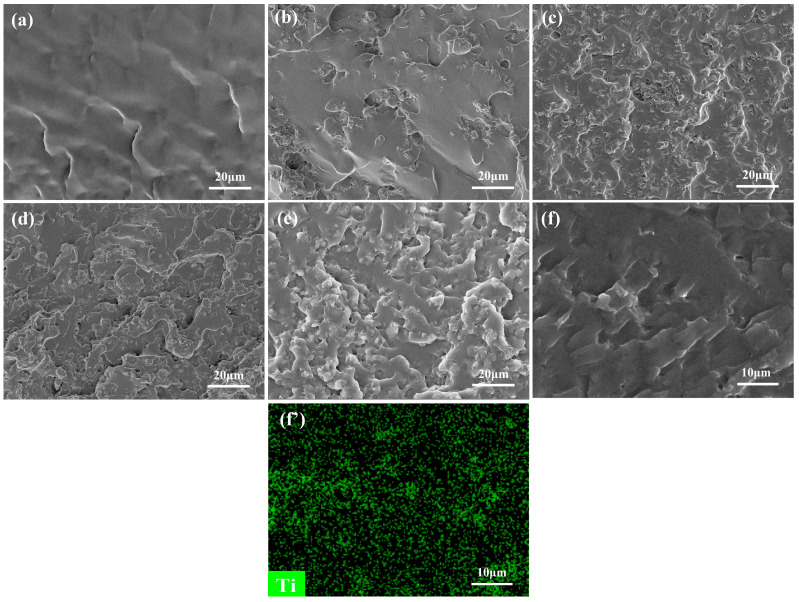
SEM images of the fractured surfaces of (**a**) WPUA/MXene-0, (**b**) WPUA/MXene-1, (**c**) WPUA/MXene-2, (**d**) WPUA/MXene-3, (**e**) and WPUA/MXene-4, (**f**,**f′**) the element-mapping images of WPUA/MXene-2.

The mechanical properties of WPUA/MXene nanocomposite films were studied through tensile testing. As observed in Figure 3, the tensile strength, the elongation at break, and Young’s modulus of the WPUA/MXene nanocomposite films exhibited a trend of initially ascending and subsequently descending as the MXene loading increased. In comparison to the WPUA/MXene-0 film, the WPUA/MXene-1 sample exhibited a 39.9% increase in tensile strength and a 38.5% increase in elongation at break. This phenomenon can be mainly attributed to two factors. Firstly, when the MXene content is relatively low, it facilitates the effective dispersion of MXene within the polymer matrix. Secondly, the terminal groups of MXene have the capability to form connections with functional groups (such as –OH, =O, –COO, and –NH–) present on the WPUA segments. These connections are formed through hydrogen bonding and van der Waals forces [29], which help maintain the crosslinking network structure of the polymer matrix. Furthermore, the high surface area of MXene plays a significant role in dispersing stress and impeding crack propagation during the stretching process, leading to improved mechanical properties of the film. Nevertheless, the tensile strength, elongation at break, and Young’s modulus of the nanocomposite films encountered a decline with additional augmentation in the MXene content. To explain this phenomenon, further investigation was conducted by SEM characterization and energy-dispersive spectroscopy (EDS) analysis of the fracture surface of the film, to study the dispersion morphology of MXene within the WPUA matrix and its interface interactions with the matrix. The WPUA/MXene-0 film exhibited a smooth fracture surface (Figure 4a). For the WPUA/MXene-1 film, the fracture surface exhibited the presence of rough areas, as shown in Figure 4b, which corresponded to the interface between the polymer matrix and MXene nanosheets. These interfaces effectively dispersed stress and hindered crack propagation upon fracture. Element-mapping images (Figure 4f,f′) revealed that in the WPUA/MXene-2 sample, the representative element Ti of MXene was uniformly distributed but more densely concentrated in the rough areas, indicating the presence of MXene agglomerates in these regions. As the MXene content continued to increase, the fracture surface of the film became rougher, suggesting an increased quantity of agglomerates. The presence of MXene agglomerates made it easier for stress concentration to occur during stretching, leading to a decrease in tensile strength. Moreover, the excessive addition of MXene disrupted the crosslinking network structure of the polymer matrix, resulting in a decrease in polymer stiffness, which further deteriorated the tensile strength and Young’s modulus. It is worth noting that the excessive addition of MXene leads to a slight decrease in fracture elongation, but it is still higher than that of WPUA/MXene-0. This is mainly due to the agglomeration of MXene, which reduces the interface area between MXene and the polymer matrix, thereby decreasing the interfacial bonding strength and affecting the load transfer and stress distribution, resulting in a decrease in the strain at break. Additionally, the agglomeration of MXene also leads to a decrease in the crosslinking density of the polymer, which makes it easier for polymer chains to undergo relative displacement and deformation, thereby increasing the strain at break.

### 3.5. Thermal Properties of WPUA/MXene Nanocomposite Films

**Figure 5:**
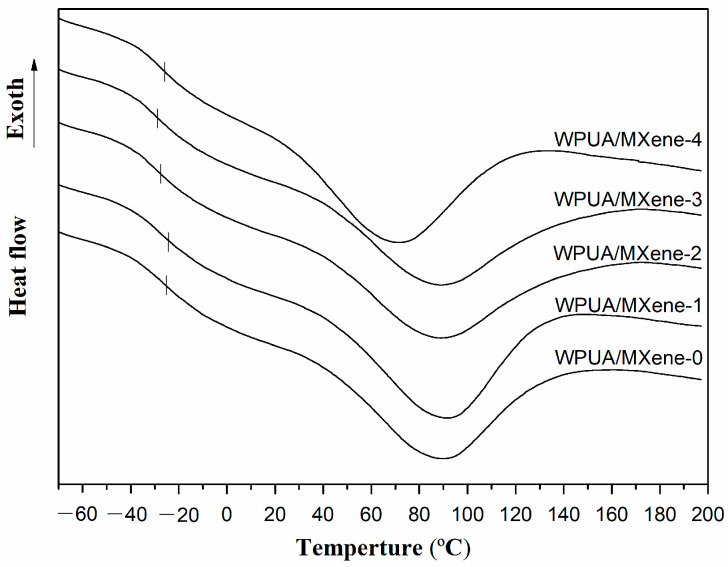
DSC curves of the WPUA/MXene nanocomposite films.

**Figure 6:**
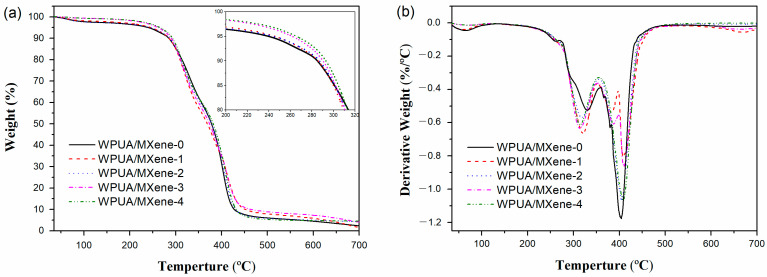
(**a**) TGA and (**b**) DTG curves of the WPUA and WPUA/MXene nanocomposites under a nitrogen atmosphere.

DSC (Figure 5) and TGA (Figure 6) were employed to investigate the thermal properties of WPUA/MXene nanocomposite films. The corresponding thermal data can be found in Table 2. From the DSC results, the glass transition temperature (T_g_) of WPUA/MXene-0 was –24.9 °C, while the T_g_ of the WPUA/MXene-1 film (–24.3 °C) increased by 0.6 °C compared to WPUA/MXene-0. This increase is mainly attributed to the effective dispersion of MXene within the polymer matrix at lower MXene content. The well-dispersed MXene does not impact the crosslink density of WPUA but effectively hinders the segmental movement of WPUA, resulting in an elevated T_g_. However, continuing to increase the MXene loading, the T_g_ of the WPUA/MXene nanocomposite films significantly decreases. This is mainly due to the increased agglomeration of MXene, which disrupts the crosslinking network of WPUA and leads to a decrease in the crosslink density.

Figure 6a,b displays the TGA and derivative thermogravimetric (DTG) curves of the WPUA/MXene nanocomposite films, respectively. As shown in Figure 6b, WPUA/MXene-0 exhibits three distinct weight loss processes. The decrease in weight observed below 130 °C can be attributed to the evaporation of moisture present in the WPUA film. The weight loss between 270 and 350 °C primarily results from the decomposition of WPUA side chains. The weight loss at around 400 °C is mainly due to the decomposition of the WPUA main chains. For the WPUA/MXene nanocomposite films, the weight loss below 130 °C decreased compared with WPUA/MXene-0, indicating a lower moisture content in the sample. According to Table 2, the temperature corresponding to a weight loss of 5 wt% (T_5%_) generally increases with increasing MXene loading. For instance, the T_5%_ of the WPUA/MXene-4 film (265.8 °C) is 26.2 °C higher than that of the WPUA/MXene-0 film (239.6 °C). When the temperature exceeds 300 °C, compared to the WPUA/MXene-0 film, the degradation rate of the WPUA/MXene nanocomposite films rapidly increases. Additionally, the temperature at the maximum decomposition rate (T_max1_) gradually decreases with increasing MXene content, which is mainly because MXene reduces the crosslink density of WPUA; as a result, apart from side chains, some small molecular segments of WPUA will also decompose first. Around 400 °C, the degradation rate of the WPUA/MXene nanocomposite films significantly slows down, and the temperature at the maximum decomposition rate (T_max2_) is higher compared to the WPUA/MXene-0 film. This is mainly attributed to the physical barrier effect of MXene, which can restrict the movement of the WPUA main chains to some extent and reduce thermal diffusion during the degradation process [16,30].

## 4. Conclusions

In this study, UV-curable WPUA/MXene nanocomposite films were prepared. The FTIR results indicated that MXene did not affect the conversion of C=C in the photopolymerization reaction, ensuring complete photopolymerization. Due to the good hydrophilicity of MXene nanosheets, the hydrophilicity of the WPUA/MXene nanocomposite films was significantly improved, with the water contact angle decreasing from 82.5° for pure WPUA to 44.4° for the WPUA/MXene-4 sample.

When the MXene content is relatively low, uniformly dispersed MXene not only does not disrupt the crosslinked network of the polymer but also effectively disperses stress during the stretching process and hinders crack propagation. As a result, the WPUA/MXene-1 film exhibited the highest tensile strength and elongation at break. 

Compared to WPUA/MXene-0, the T_g_ of the WPUA/MXene-1 film increased by 0.6 °C, indicating an improved crosslinking density. However, the subsequent increase in the MXene content reduced the crosslinking density, resulting in a decrease in T_g_. TGA analysis revealed that the addition of MXene increased both the T_5%_ and T_max2_ in the nanocomposite films. 

This study introduces MXene, which exhibits excellent dispersibility in water, into a waterborne UV-curable coating system known for its environmental friendliness and high efficiency. As a result, the prepared WPUA/MXene nanocomposite coatings demonstrate excellent hydrophilicity, enhanced mechanical properties, and improved thermal stability. This work lays the foundation for the application of MXene in the field of UV-curable coatings. In the future, if the photo-initiation efficiency of the system can be further improved to prepare films with a higher content of MXene, it would enable the WPUA/MXene nanocomposite coatings to have a wider range of applications in areas such as conductive coatings and electromagnetic shielding.

## Figures and Tables

**Table 1:** Formulations of WPUA/MXene nanocomposite coatings.

Sample	MXene Content/wt%	UV-Curable WPUA Mixture/g	MXene Solution/mL	H_2_O/mL
WPUA/MXene-0	0	30	0	20
WPUA/MXene-1	0.077	30	5	15
WPUA/MXene-2	0.154	30	10	10
WPUA/MXene-3	0.231	30	15	5
WPUA/MXene-4	0.308	30	20	0

**Table 2:** TGA data of the WPUA and WPUA/MXene nanocomposites under a nitrogen atmosphere.

Sample	T_g_ (°C)	T_5%_ (°C)	T_max1_ (°C)	T_max2_ (°C)
WPUA/MXene-0	−24.9	239.6	329.7	404.3
WPUA/MXene-1	−24.3	245.6	320.8	407.6
WPUA/MXene-2	−27.7	244.2	318.3	406.1
WPUA/MXene-3	−28.9	261.6	314.6	409.8
WPUA/MXene-4	−26.0	265.8	313.8	408.6

## Data Availability

Data are contained within the article.
